# The Relationship between Alcohol Consumption and Incidence of Glycometabolic Abnormality in Middle-Aged and Elderly Chinese Men

**DOI:** 10.1155/2016/1983702

**Published:** 2016-02-14

**Authors:** Siwen Zhang, Yujia Liu, Gang Wang, Xianchao Xiao, Xiaokun Gang, Fei Li, Chenglin Sun, Ying Gao, Guixia Wang

**Affiliations:** Department of Endocrinology and Metabolism, The First Hospital of Jilin University, No. 8 Xinmin Street, Changchun, Jilin 130021, China

## Abstract

*Aim.* The relationship between alcohol consumption and glycometabolic abnormality is controversial, especially in different ethnic population. In this study, a cross-sectional survey was carried out to examine the relationship between alcohol consumption and glycometabolic abnormality in middle-aged and elderly Chinese men.* Methods.* Using cluster random sampling, Chinese men aged more than 40 years from Changchun, China, were given standardized questionnaires. In total, 1996 individuals, for whom complete data was available, were recruited into the study. We calculated the incidence of prediabetes and newly diagnosed diabetes by three levels of alcohol consumption: light, moderate, and heavy. Multivariate logistic regression models adjusted for socioeconomic variables and diabetes-related risk factors were used to analyze the association between alcohol consumption and the onset of prediabetes and diabetes.* Results.* The univariate analysis revealed higher incidence of prediabetes among drinkers (32.8%) compared with nondrinkers (28.6%), particularly in heavy alcohol consumers. The logistic regression analysis showed that alcohol consumption, especially heavy consumption, was an independent risk factor for prediabetes.* Conclusions.* Alcohol consumption, heavy consumption in particular, is an independent risk factor for the development of prediabetes, but not for diabetes.

## 1. Introduction

Diabetes mellitus is a group of metabolic disorders with phenotypic features similar to those of hyperglycemia. As a chronic condition, diabetes can cause serious complications such as cardiovascular, gastrointestinal, and genitourinary disease and nephropathy, which are major direct causes of diabetes-related deaths [1]. Diabetes has become a major global public health problem and the global burden of diabetes mellitus has been steadily increasing. The onset of type 2 diabetes mellitus (T2DM) is closely associated with diet and lifestyle, especially in people who are prediabetic.

Alcohol consumption, a common social custom in most parts of the world, has been reported to be associated with diabetes onset in numerous observational studies [2]. Multiple studies have investigated the association between alcohol consumption and prediabetes and diabetes, and the results are controversial. A prospective study by Valmadrid et al. indicated that alcohol consumption increases the risk of diabetes [3]. Koppes et al. in a metaregression analysis of 15 prospective studies reported that the relative risk (RR) of T2DM differs with the level of alcohol consumption. Moderate alcohol consumers (6–48 g/day) have a 30% decreased RR of type 2 diabetes compared to nondrinkers [4]. Some epidemiological studies have shown a J- or U-shaped relation between alcohol consumption and T2DM [5]. Although alcohol consumption is common in Chinese culture, people are still unaware of its effects on health. The Chinese National Diabetes Epidemiology Investigation in 2011 revealed that the occurrences of alcohol consumption and diabetes are quite high (38.1%, 6.4%) [6]; therefore, in this study we included participants who were diagnosed with diabetes or prediabetes during the study protocol and excluded those men who have been previously diagnosed with diabetes. We aimed to examine the association between alcohol consumption and glycometabolic abnormality to aid development of prevention strategies and intervention measures for diabetes.

## 2. Methods

The present work is a part of the baseline survey for the Risk Evaluation of Cancers in Chinese Diabetic Individuals: a Longitudinal (REACTION) study, conducted among 259,657 adults, aged ≥40 years from 25 communities across mainland China, from 2011 to 2012 [7–10].

### 2.1. Population Selection

Using cluster random sampling via field investigation, we selected residents (dwelling for at least 5 years or more) from 20 communities, including those of Changchun and neighboring communities, who aged more than 40 years. A total of 1996 individuals were successfully recruited into the present study; the response rate was 20.9%. Each participant was provided with an informed consent. The procedures used in this study were approved by the local ethics committee of Jilin University, China, and conformed to the provisions of the Declaration of Helsinki (as revised in Seoul, 2008).

### 2.2. Exclusion Criteria

Individuals with any of the following conditions were excluded from this study: (1) history of diabetes, (2) recent history of fever, (3) history of cardiovascular disease, (4) acute stress reactions such as infection, surgery, or trauma, and (5) pregnancy.

### 2.3. Content of Inquiry

#### 2.3.1. Questionnaire Survey

All participants were interviewed by trained physicians or public health workers. Data regarding socioeconomic condition, lifestyle, and health status were collected using standardized questionnaires containing questions on age, sex, race, marital history, reproductive history, level of education, occupation, cigarette smoking, history and treatment of diabetes, physical activity, alcohol consumption-related information including whether currently drinking or not, years of drinking, categories of alcohol beverages (white spirit, beer, claret, and rice wine), and amount of alcohol consumption per day.

#### 2.3.2. Physical Assessments

Blood pressure (BP), pulse, height, weight, waist circumference, and hip circumference were included. BP was measured three times consecutively, with an Omron blood pressure monitor, with an interval of 1 min, and the average of three tests was recorded as the baseline BP. The measurements of height and weight were conducted using Overlord devices.

Fasting venous glucose, hepatic function test, renal function test, blood lipid levels, and glycosylated hemoglobin (HbA1C) test of participants are measured. Participants without diabetes were tested with oral glucose tolerance test (OGTT), and for newly diagnosed diabetic subjects, venous glucose concentration was obtained 2 hours after the intake of 100 g of steamed bun. HbA1C was measured with High Performance Liquid Chromatography (HPLC), variant II glycosylated hemoglobin detectors, American Bole Company.

### 2.4. Diagnostic Criteria

Criteria of glycometabolism categories are as follows [11]:   (1) normal glucose regulation (NGR): FBG < 6.1 and 2hPBG < 7.8; (2) prediabetes: I: impaired fasting glucose (IFG): FBG: 6.1–7.0 and 2hPBG < 7.8; II: impaired glucose tolerance (IGT): FBG < 7.0 and 2hPBG: 7.8–11.1; and (3) DM: FBG ≥ 7.0 or 2hPBG ≥ 11.1 (FBG: Fasting Blood Glucose, mmol/L; 2hPBG: 2-Hour Postprandial Blood Glucose, mmol/L).

According to the criteria of 2010 Chinese guidelines for the management of hypertension, hypertension can be diagnosed based on at least one of the following items: (1) systolic pressure (SP) ≥ 140 mmHg; (2) diastolic pressure (DP) ≥ 90 mmHg; and (3) previous hypertension.

According to the recommendations of the Working Group on Obesity in China, 2002, (1) overweight is 24.0 kg/m^2^ ≤ body mass index (BMI) < 28.0 kg/m^2^ and (2) obesity is BMI ≥ 28.0 kg/m^2^.

### 2.5. Definition of Relative Risk Factors

Alcohol consumption assessment is as follows: (1) nondrinker: never consumed or consumed alcohol occasionally; (2) drinker: consumed alcohol (any type) at least once a week; (1) light alcohol consumption: amount < 30 g/d; (2) moderate alcohol consumption: 30 g/d ≤ amount < 50 g/d; and (3) heavy alcohol consumption: amount ≥ 50 g/d (amount [g/d] = daily alcohol volume [mL] *∗* alcoholicity [V/V] *∗* density [g/mL]; density of alcohol was calculated as 0.8 g/mL) [12].

### 2.6. Cigarette Smoking Assessment

Participants who smoked at least one cigarette per day or seven cigarettes per week for more than 6 months were defined as “smokers.” Individuals who had never smoked or smoked occasionally were defined as “nonsmokers.”

### 2.7. Statistical Methods

Results of questionnaire and laboratory testing were fed into EXCEL to set up a database to be used for data analysis. Descriptive and comparable statistical analyses between “drinker” and “nondrinker” groups with the parameters age, BMI, waist-hip ratio (WHR), hepatic function, renal function, FBG, 2hPBG, and HbA1C were conducted. Statistics were generated with SPSS (version 18.0, SPSS Inc., IBM, Armonk, NY, USA). Measurement data were expressed as mean ± SD. Disease risk was evaluated as odds ratio (OR). Univariate analysis was conducted with chi-square test. The average levels of two groups were measured with *t*-test. Multivariate logistic regression models adjusted for confounding factors were used to examine ordinal variable.

## 3. Results

**Figure 1:**
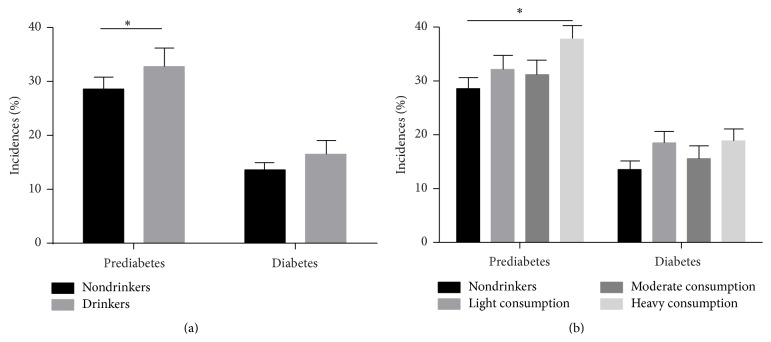
(a) The incidence of prediabetes and diabetes in group of drinkers and nondrinkers. 28.6% and 32.8% of drinkers showed prediabetes and diabetes; 13.6% and 16.5% of nondrinkers showed prediabetes and diabetes. (b) The incidence rates of prediabetes and diabetes in the case of none, light, moderate, and heavy alcohol consumption.

The basic information of participants is that a total of 1996 men aged more than 40 years were examined. Baseline demographic and other characteristics of the study participants are presented in Table 1. The mean age of the participants was 57.70 years (57.7 ± 10.60 years) and 31.96% of participants consumed alcohol. Of all participants, 14.49% were newly diagnosed with T2DM and 29.94% with prediabetes. On comparing the incidence of newly diagnosed diabetes and prediabetes (IFG or IGT) in drinkers and nondrinkers (Figure 1(a)), incidence of prediabetes was higher among drinkers than among nondrinkers. There was no statistical significance except for the difference between heavy drinkers and nondrinkers in the incidence of prediabetes (Table 2 and Figure 1(b)). In all age groups after age stratification, there is no statistically significant difference between drinkers and nondrinkers in the incidences of diabetes when assessed using the chi-square test (see Supplementary Table  1, Supplementary Figure  1 in Supplementary material available online at http://dx.doi.org/10.1155/2016/1983702).

Multivariate logistic regression models adjusted for socioeconomic variables and diabetes-related risk factors including age, level of education, BMI, smoking habits, drinking status, family history, physical activity, calorie intake, systolic BP, and high density lipoprotein (HDL) were used to analyze the contribution of alcohol in the onset of prediabetes and diabetes. With drinking (including light, moderate, and heavy amounts) as a single variable, the results showed that “age,” “drinking,” and “BMI” were independent risk factors for prediabetes. “Age” and “BMI” were independent risk factors for diabetes as well. However, “drinking” was not an independent risk factor for diabetes since the *P* value of factor “drinking” was 0.1 (>0.05) (Tables 3 and 4).

With light, moderate, and heavy alcohol consumption as three separate variables, according to the diagnostic criteria and the definition of risk factors as described in “Section 2,” the factors “age,” “drinking,” “level of education,” and “BMI” were analyzed as categorical variables. The results showed that “age (50–59),” “age (60–69),” “age (>70),” “heavy consumption of alcohol,” and “obesity” were independent risk factors for prediabetes. “Age (50–59),” “age (60–69),” “age (>70),” “overweight,” and “obesity” were independent risk factors for diabetes (Tables 5 and 6).

## 4. Discussion 

The relationship between diabetes and alcohol consumption is complex. Although heredity is an established risk factor for type 2 diabetes, personal lifestyle is important and crucial in the etiology. Other factors, including overweight and lack of physical activity, also increase risk for T2DM. There is growing consensus that alcohol consumption is another important contributor. Epidemiology studies have shown a J-shaped relation between alcohol consumption and incidence of T2DM [13], while others have reported a U-shaped relation, indicating moderate alcohol consumption is protective against diabetes, which raises the question of the effect of higher amount of alcohol consumption in the development of T2DM. In addition, other researchers reported that alcohol consumption increased the chance of developing diabetes, including the risk of moderate consumption [14]. Nonetheless, these reviews were not able to provide a definitive conclusion about the relationship between alcohol consumption and glycometabolic abnormality [15].

In addition, the association mentioned above was not found in all racial and ethnic groups. Few studies systematically evaluated this relationship of alcohol consumption and glycometabolic abnormality in Chinese population. Therefore, we aimed to investigate the relationship between alcohol consumption with prediabetes and DM among middle-aged and elderly Chinese men. Since women usually do not regularly consume alcohol, Chinese men were evaluated in this study. Epidemiological data were collected by the research of Chinese Medical Association at Changchun in 2011, and a standard control was used during the investigation process, laboratory examination, data exclusion, and statistical analysis. A total of 1996 cases were recruited to evaluate the influence of alcohol on the incidence of prediabetes and diabetes in elderly Chinese men. It is noted that the participants diagnosed with diabetes previously were excluded from the study to improve reliability of our research since these subjects could have been motivated to abstain from alcohol or change their lifestyles due to health concerns. They could have confounded the risk of developing diabetes, known as the sick-quitter effect [16].

In our study, the drinking rate of participants with prediabetes and diabetes was higher than of those with normal blood glucose levels. The incidence of prediabetes was higher among alcohol drinkers than among nondrinkers, and “drinking” was an independent risk for prediabetes. When analyzing light, moderate, and heavy alcohol consumption, only heavy daily alcohol consumption was associated with the risk of prediabetes in participants using chi-square test. Multivariate logistic regression analysis also supported that result. However, there is no certain correlation between daily alcohol consumption and incidence of diabetes. We infer that drinking, especially heavy alcohol consumption, may increase the incidence of prediabetes in elder Chinese men. However, these findings are absent when analyzing the incidence of diabetes even though the incidence of diabetes had a similar trend to that of prediabetes. Furthermore, the result after age stratification showed no statistically significant difference between drinkers and nondrinkers in both prediabetes and diabetes. These findings were somewhat different from those of prior studies conducted in Guangdong province, China [17]. They found a J-shape relation between alcohol consumption and incidence of diabetes and insulin-like growth factor-I (IGF). Furthermore, multivariate logistic regression analysis showed that age and BMI were two other independent risk factors for prediabetes and diabetes. The risk may increase 0.028 (prediabetes) and 0.038 (diabetes) times with 1-year increase in age. The risk may increase 0.066 (of prediabetes) and 0.145 (of diabetes) times with the 1 kg/m^2^ BMI increase. These findings were somewhat similar to those of Janghorbani et al. [18].

In general, the aforementioned results indicate that the occurrence of prediabetes could be stimulated by alcohol, especially heavy daily alcohol consumption, but there is no correlation between alcohol consumption and the incidence of diabetes. On the other hand, individuals with prediabetes may change their lifestyle, for example, abstinence. This behavior may affect the research results and increase the deviation. Based on our research, we believe that alcohol may not have a definitive relationship with the onset and progression of diabetes. To further confirm the relationship between alcohol drinking and diabetes, enlarging the sample size or excluding individuals with prediabetes history could be a good approach.

The impact of alcohol on glycometabolism is uncertain in terms of biological mechanism, but there are several factors that may explain this relationship. Alcohol may have positive effects, such as protecting against diabetes by increasing insulin sensitivity [19], changing levels of alcohol metabolites [20], increasing HDL concentrations [21], or the anti-inflammatory effect of alcohol [22]. Crandall et al. showed that higher alcohol consumption was associated with lower insulin secretion at any level of insulin sensitivity [23]. Alcohol may also have the negative effects; it is well established that excessive alcohol consumption can lead to various liver diseases as well as chronic or acute pancreatitis [24]. Furthermore, Liang and Chikritzhs indicated that associations between drinking and the risk of diabetes exist due to genetic predisposition, rather than the protective effects of alcohol itself [25]. Some researchers found that alcohol facilitated the development of T2DM by increased uptake of glycolipids [26]. Hormones such as IGF-I and growth hormone (GH) are prominent in defining the risk and development of T2DM and may be adversely affected by heavy alcohol use, possibly mediating its diabetogenic effects [12]. On the other hand, the overlaps of different factors could increase the deviations of statistical analysis: for example, diabetic patients who are alcohol consumers are likely to have poor adherence for diabetic treatment and are associated with increased morbidity and mortality [27]. Engler et al. believed that alcohol affects judgment, such as decreased attention to diet and medication, and may also impair other self-care behaviors such as exercise and glucose self-monitoring [27]. Thus, drinking may affect the incidence of prediabetes or diabetes indirectly by affecting lifestyle.

In conclusion, our survey indicated that alcohol can facilitate the development of prediabetes, but it is not an independent risk factor for diabetes, even with heavy drinking. However, alcohol drinking, especially heavy consumption, can increase the risk of prediabetes. Also, heavy alcohol consumption is a risk factor for both diabetes and poor treatment adherence [28]. According to the influences mentioned above, it is suggested that individuals with drinking habit should restrict the amount to light or moderate level.

## 5. Conclusion

Alcohol consumption, heavy consumption in particular, is an independent risk factor for the development of prediabetes, but not for diabetes.

## Supplementary Material

In age group I, II, and III, drinkers showed higher rates of pre-diabetes than nondrinkers, the difference was not statistically significant. While in age group IV, the incidence of prediabetes was higher in the nondrinkers than that of drinkers; the difference was not statistically significant either. In all age groups, drinkers had higher rates of diabetes than non-drinkers, while the difference was not statistically significant.

## Figures and Tables

**Table 1:** Baseline demographics and characteristics of participants.

	Nondrinkers	Drinkers	*P*
Participants, *n* (%)	1358 (68.03%)	638 (31.96%)	
Age^**∗**^, y	58.24 ± 11.0	56.54 ± 9.70	0.015
BMI, kg/m^2^	25.37 ± 3.29	25.54 ± 3.68	0.121
WHR	0.88 ± 0.06	0.89 ± 0.06	0.182
FPG (mmol/L)	5.87 ± 1.37	5.98 ± 1.38	0.097
2hPG (mmol/L)	7.57 ± 3.34	7.78 ± 3.48	0.182
HbA1c (%)	5.85 ± 0.79	5.85 ± 0.85	0.951
SBP^**∗**^ (mmHg)	141.84 ± 21.20	144.25 ± 20.25	0.011
DBP^**∗**^ (mmHg)	82.72 ± 11.72	84.88 ± 11.47	<0.01
ALT^**∗**^ (U/L)	16.03 ± 11.37	17.30 ± 11.77	0.022
AST^**∗**^ (U/L)	22.27 ± 9.19	25.42 ± 17.32	<0.01
GGT^**∗**^ (U/L)	34.82 ± 33.21	61.97 ± 91.08	<0.01
Family history of diabetes, *n* (%)	131 (9.92%)	57 (9.36%)	0.097
Education^**∗**^ >11 years, *n* (%)	863 (63.55%)	370 (57.99%)	<0.01
Smoking status^**∗**^ (%)	416 (31.42%)	435 (71.55%)	0.040
Regular physical activity^**∗**^, *n*, %	928 (68.34%)	392 (61.44%)	<0.01
High energy intake, *n* (%)	513 (37.78%)	246 (38.56%)	0.079

^**∗**^There is a statistical significant difference between drinkers and nondrinkers (*P* < 0.05).

**Table 2:** The incidence of newly diagnosed diabetes and prediabetes in the drinkers and nondrinkers (%).

	All	Nondrinkers	Light consumption	Moderate consumption	Heavy consumption
Normal	1109	785 (39.33)	143 (7.16)	109 (5.46)	71 (3.56)
Prediabetes	598	388 (19.44)	89 (4.46)	61 (3.06)	60 (3.01)
DM	289	185 (9.27)	47 (2.35)	30 (1.50)	28 (1.40)

**Table 3:** Multivariate logistic regression analysis of prediabetes (drinking as a single variable).

	Beta	SE	OR (95% CI)	*P*
Age^**∗**^	0.028	0.006	1.028 (1.015–1.041)	0.0001
Drinking^**∗**^	0.280	0.139	1.322 (1.006–1.737)	0.049
Smoking	0.020	0.133	1.021 (0.787–1.324)	0.273
BMI^**∗**^	0.063	0.018	1.066 (1.028–1.105)	0.0001
Education level	0.208	0.650	1.232 (0.344–4.406)	0.805
Family history of DM	−0.150	0.205	0.861 (0.576–1.288)	0.629
Calorie intake	−0.106	0.123	0.899 (0.707–1.144)	0.462
Physical activity	0.152	0.138	1.164 (0.887–1.527)	0.337
SBP	0.004	0.003	1.004 (0.998–1.010)	0.124
HDL	−0.108	0.221	0.898 (0.582–1.384)	0.325

^**∗**^There is a statistical significant difference between drinkers and nondrinkers (*P* < 0.05).

**Table 4:** Multivariate logistic regression analysis of diabetes (drinking as a single variable).

	Beta	SE	OR (95% CI)	*P*
Age^**∗**^	0.037	0.008	1.038 (1.021–1.055)	<0.001
Drinking^**∗**^	0.333	0.183	1.396 (0.974–1.999)	0.100
Smoking	−0.053	0.177	0.948 (0.670–1.342)	0.521
BMI^**∗**^	0.136	0.024	1.145 (1.093–1.201)	<0.001
Education level	−0.916	0.560	0.340 (0.133–1.198)	0.508
Family history of DM	−0.309	0.258	0.734 (0.443–1.216)	0.146
Calorie intake	−0.204	0.163	0.815 (0.592–1.123)	0.379
Physical activity	−0.195	0.176	0.823 (0.583–1.162)	0.348
SBP	0.003	0.004	1.003 (0.995–1.011)	0.103
HDL	0.107	0.280	1.113 (0.643–1.927)	0.501

^**∗**^There is a statistical significant difference between drinkers and nondrinkers (*P* < 0.05).

**Table 5:** Multivariate logistic regression analysis of prediabetes (light, moderate, and heavy alcohol consumption as three separate variables).

	Beta	SE	OR (95% CI)	*P*
Age^*∗*^ (50–59)	0.671	0.186	1.955 (1.358–2.815)	<0.001
Age^*∗*^ (60–69)	0.810	0.195	2.247 (1.534–3.292)	<0.001
Age^*∗*^ (>70)	0.739	0.232	2.093 (1.328–3.299)	0.001
Drinking (light consumption)	0.292	0.211	1.339 (0.886–2.024)	0.512
Drinking (moderate consumption)	0.275	0.247	1.316 (0.810–2.138)	0.864
Drinking^*∗*^ (heavy consumption)	0.584	0.280	1.794 (1.035–3.108)	0.048
Smoking	−0.061	0.141	0.941 (0.714–1.241)	0.537
Overweight	0.261	0.147	1.298 (0.974–1.730)	0.100
Obesity^*∗*^	0.715	0.182	2.045 (1.430–2.923)	0.001
Education = 2	0.149	0.654	1.161 (0.322–4.187)	0.613
Education = 3	0.324	0.622	1.383 (0.409–4.677)	0.869
Education = 4	0.473	0.620	1.605 (0.476–5.415)	0.902
Education = 5	0.198	0.622	1.219 (0.360–4.129)	0.988
Family history of DM	−0.081	0.218	0.923 (0.601–1.415)	0.402
Calorie intake	−0.133	0.130	0.876 (0.678–1.130)	0.331
Physical activity	0.082	0.146	1.086 (0.815–1.447)	0.214
SBP	0.003	0.004	1.003 (0.995–1.011)	0.129
HDL	0.107	0.280	1.113 (0.643–1.927)	0.301

Education: 1 = illiteracy, 0 years of education; 2 = elementary school level, 6 years of education; 3 = middle school level, 9 years of education; 4 = high school level, 12 years of education; 5 = university level or higher, 16 years of education or higher.

^*∗*^There is a statistical significant difference between drinkers and nondrinkers (*P* < 0.05).

**Table 6:** Multivariate logistic regression analysis of diabetes (light, moderate, and heavy alcohol consumption as three separate variables).

	Beta	SE	OR (95% CI)	*P*
Age^*∗*^ (50–59)	0.805	0.247	2.237 (1.379–3.631)	0.001
Age^*∗*^ (60–69)	0.691	0.266	1.995 (1.185–3.360)	0.001
Age^*∗*^ (>70)	1.061	0.296	2.888 (1.617–5.160)	<0.001
Drinking (light consumption)	0.449	0.271	1.567 (0.921–2.666)	0.100
Drinking (moderate consumption)	0.424	0.324	1.527 (0.810–2.881)	0.341
Drinking^*∗*^ (heavy consumption)	0.406	0.388	1.501 (0.702–3.209)	0.623
Smoking	−0.255	0.190	0.775 (0.534–1.126)	0.316
Overweight^*∗*^	0.662	0.209	1.939 (1.287–2.922)	0.001
Obesity^*∗*^	1.130	0.243	3.093 (1.920–4.984)	<0.001
Education = 2	−1.273	0.739	0.280 (0.066–1.192)	0.328
Education = 3	−0.604	0.638	0.547 (0.156–1.910)	0.299
Education = 4	−0.570	0.637	0.565 (0.162–1.969)	0.375
Education = 5	−0.617	0.639	0.540 (0.154–1.887)	0.422
Family history of DM	−0.141	0.277	0.868 (0.504–1.494)	0.397
Calorie intake	−0.190	0.173	0.827 (0.589–1.162)	0.212
Physical activity	−0.125	0.186	1.086 (0.612–1.272)	0.410
SBP	0.003	0.004	1.003 (0.995–1.011)	0.131
HDL	0.107	0.280	1.113 (0.643–1.927)	0.224

Education: 1 = illiteracy, 0 years of education; 2 = elementary school level, 6 years of education; 3 = middle school level, 9 years of education; 4 = high school level, 12 years of education; 5 = university level or higher, 16 years of education or higher.

^*∗*^There is a statistical significant difference between drinkers and nondrinkers (*P* < 0.05).
