# Supplementary material for: The Relationship between Alcohol Consumption and Incidence of Glycometabolic Abnormality in Middle-Aged and Elderly Chinese Men
Source: Int J Endocrinol. 2016 Feb 14;2016:1983702. doi: 10.1155/2016/1983702 (PMC4769752; doi:10.1155/2016/1983702)

**Figure legends:**

**Supplementary Fig.1** the participants were divided into four age groups: 40-49 years; 50-59 years; 60-69 years; over 70 years. The incidence of prediabetes and diabetes among drinkers and nondrinkers in different age groups was compared.

**Supplementary table 1**

Characteristics of participants blood glucose in different age group (％)

| Age group | Blood glucose status | Nondrinkers | Drinkers | p |
| --- | --- | --- | --- | --- |
| 40-49  (I) | Normal | 208 (10.42) | 92 (4.61) | 0.11 |
|  | Pre-diabetes | 46 (2.3) | 66 (3.31) | 0.10 |
|  | DM | 19 (0.95) | 35 (1.75) | 0.26 |
| 50-59  (II) | Normal | 250 (12.52) | 118 (5.91) | 0.08 |
|  | Pre-diabetes | 79 (3.96) | 117 (5.86) | 0.15 |
|  | DM | 46 (2.30) | 59 (2.96) | 0.14 |
| 60-69  (III) | Normal | 210 (10.52) | 80 (4.01) | 0.13 |
|  | Pre-diabetes | 65 (3.26) | 132 (6.61) | 0.17 |
|  | DM | 28 (1.40) | 53 (2.669) | 0.20 |
| >70  (IV) | Normal | 114 (5.71) | 32 (1.60) | 0.24 |
|  | Pre-diabetes | 20 (1.00) | 75 (3.76) | 0.21 |
|  | DM | 13 (0.65) | 39 (1.95) | 0.19 |

Supplementary figure 1.


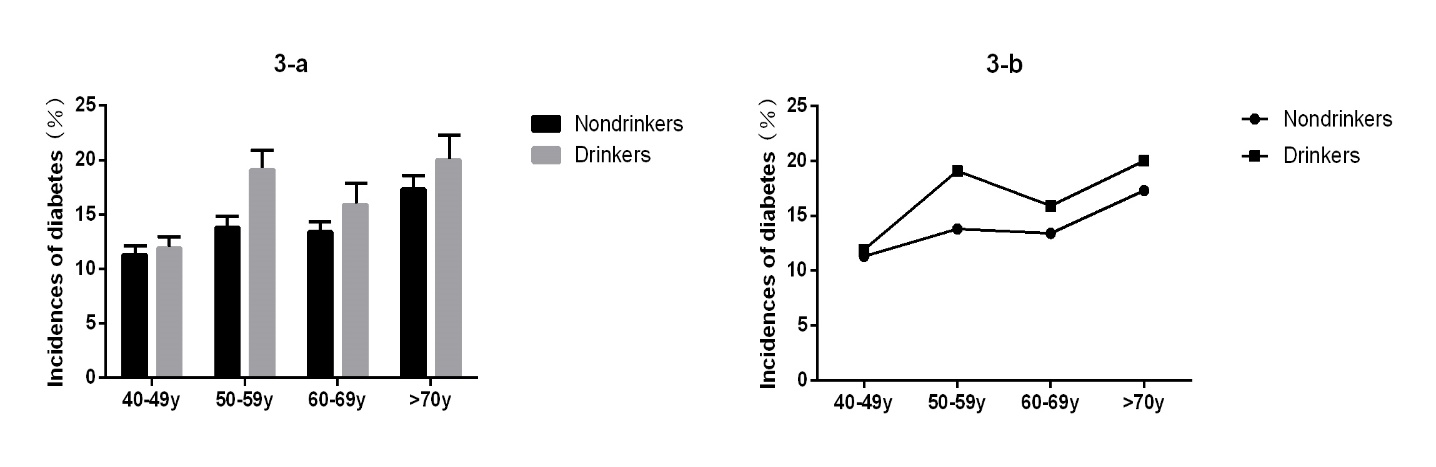

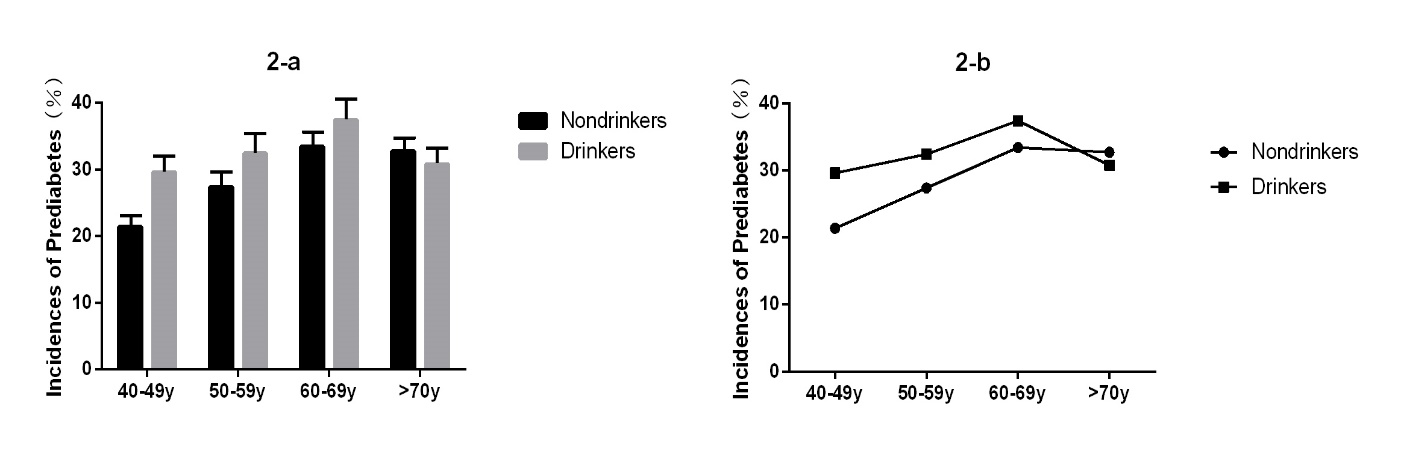

Supplement: Supplementary file 1 — In age group I, II, and III, drinkers showed higher rates of pre-diabetes than nondrinkers, the difference was not statistically significant. While in age group IV, the incidence of prediabetes was higher in the nondrinkers than that of drinkers; the difference was not statistically significant either. In all age groups, drinkers had higher rates of diabetes than non-drinkers, while the difference was not statistically significant. [file 1983702.f1.docx]
